# School achievement in the Brazilian rural Amazon: An analysis of the interaction among motor, cognitive and environmental factors

**DOI:** 10.1111/bjdp.70035

**Published:** 2026-01-26

**Authors:** Douglas Vieira, Cinthia Rodrigues Barros, Mabliny Thuany, Thayse Natacha Gomes

**Affiliations:** ^1^ Centre of Education, Humanities and Health (CEHS) Federal University of Northern Tocantins (UFNT) Tocantinópolis Tocantins Brazil; ^2^ Postgraduate Program in Science and Mathematics Teaching Federal University of Northern Tocantins (UFNT) Araguaína Tocantins Brazil; ^3^ Department of Sports University of the State of Pará (UEPA) Tucuruí Pará Brazil; ^4^ Department of Physical Education Federal University of Sergipe São Cristóvão Sergipe Brazil

**Keywords:** academic performance, attention, network analysis, physical fitness

## Abstract

A cross‐sectional study of 106 students (7–12 years) from a rural school in the Brazilian Amazon investigated the interaction among motor, cognitive and environmental factors on school achievement using network analysis. Students with good school performance were significantly older and showed superior global physical fitness, motor coordination (balance and lower limbs) and alternating selective attention. Furthermore, students residing more than 1000 m from the school showed significantly higher overall school achievement. Network analysis identified motor coordination as an important central node for the group with insufficient achievement, suggesting motor interventions can be effective strategies for this population. For high‐achieving students, age emerged as the most central variable. The findings underscore that academic success is a complex system, with different central factors depending on the student's performance level.

**Key points**

- Students with good school achievement were significantly older and demonstrated superior global physical fitness, motor coordination (specifically balance and lower limb coordination), and alternating selective attention.
- Contrary to general assumptions, students residing more than 1000 m from the school showed significantly higher overall school achievement compared to those living closer to the village core.
- Network analysis identified motor coordination as the most important central node for the group with insufficient achievement, suggesting that motor interventions could be effective strategies to improve academic outcomes in this population.
- For high‐achieving students, age emerged as the most central variable, reflecting a developmental cascade where maturation impacts various motor and cognitive skills over time.

## INTRODUCTION

In Brazil's Northern region, which includes a significant part of the Amazon, over 65% of municipalities are classified as rural, a proportion much higher than in other parts of the country. Although the rural population accounts for only 34% of the Northern region, this substantial number raises concerns about educational access and school achievement (Agência Brasil, 2017). According to UNICEF, students in the Amazon and other Brazilian rural areas are at a high risk of educational exclusion and learning deficits, often because they need to work to support their families (United Nations, 2020).

Among rural residents, there are higher rates of educational delays and lower school achievement when compared to their urban counterparts (Nascimento & Andrade, 2022; Rodrigues et al., 2020). However, studies on the factors affecting school achievement often overlook key geographical influences, leading to the marginalization of social issues that contribute to these poor educational outcomes (Araujo & Silveira Neto, 2021; García‐Hermoso et al., 2017; Lopes et al., 2020; Nascimento & Andrade, 2022; Rodrigues et al., 2020).

In a broader perspective, school achievement is the result of a harmonious set of factors that, in addition to cognitive aspects, also include social and emotional elements. These variables can be assessed through standardized instruments to observe how they interact and influence a child's performance in school (Castro et al., 2021). Recent studies have shown that school achievement, in general, can be influenced by various factors related to the individual, such as gender and age, physical‐motor aspects (Geertsen et al., 2016; Greeff et al., 2018), learning difficulties (Izidoro et al., 2014), concentration and attention deficits (Slattery et al., 2022), and the environment, such as the place of residence and school characteristics (Nascimento & Andrade, 2022; Rodrigues et al., 2020), in which the child and the school are located.

Given its multi‐faceted nature, influenced by various individual and environmental determinants, and the interactions among these factors, school achievement should be understood as a complex system (Behringer et al., 2022; Fernandes & Lemos, 2020; Hossain et al., 2021). A system is considered complex when it is composed of numerous interacting, interdependent elements whose combined behaviour cannot be predicted from the properties of the individual components alone. This perspective aligns with the ‘Ecological Theory of Human Development’, specifically the Process‐Person‐Context‐Time (PPCT) model, which posits that a child's development, including school achievement, is influenced by the ongoing processes of interaction between the individual (e.g. age, motor skills) and the environmental contexts (e.g. school, community) (Bronfenbrenner, 2011). Therefore, understanding these agents in isolation, although important, is insufficient; it is the intrinsic interactions and interdependencies that produce emergent changes in the subject of study (Furtado et al., 2015).

Therefore, this study will investigate the factors influencing the school achievement of Amazonian Brazilian children, considering both the environment and the individual, as well as their interactions using network analysis. From an environmental perspective, we will analyse the characteristics of the school location, particularly the rural context, and the distance between the child's residence and the educational institution. From the individual perspective, we will address sociodemographic factors (age and gender), cognitive factors (specifically attention), behavioural factors (physical activity and fitness) and the child's motor skills.

Additionally, the specific determinants of success for children in rural contexts can vary by region (García‐Hermoso et al., 2017; Lopes et al., 2020). While factors such as socioeconomic status and family structure are recognized as fundamental, distal determinants of educational outcomes, the existing literature has historically prioritized purely cognitive factors, thereby neglecting the crucial and often proximal roles of behavioural and motor functions (Barbosa et al., 2020). This exclusion, particularly in studies in rural contexts and in the Brazilian Amazon, limits the understanding of the complex network of predictors.

The motivation for exploring the geographical factor in the Brazilian Amazon context stems from its unique structural characteristics. The study location has a single community structure, originating from its historical status as a resettlement area following the construction of a national hydropower plant (Sousa, 2021). This regional and historical context results in a specific array of logistical and resource‐based barriers, such as long travel distances, dependence on school transport and limited access to structured leisure areas that are hypothesized to interfere with school achievement, physical activity and attention levels (Sousa, 2021). By focusing on this geographical and historical specificity, this study aims to overcome the marginalization of these local issues and provide highly context‐specific insights into the mechanism of academic success.

Thus, the choice to emphasize motor skills, physical fitness and attention is therefore driven by the critical need to: (i) address this literature gap by focusing on physical and motor domains that are closely linked to executive brain functions critical for learning (Fernandes et al., 2016); and (ii) identify modifiable factors that can serve as feasible and impactful targets for school‐based interventions (Vilella‐Cortez et al., 2019) in this unique rural setting. By investigating the interactions among these three domains and the environment, we aim to provide a more comprehensive and integrated understanding of the complex system determining academic success.

Therefore, the purpose of this study is to analyse how these interconnected factors influence the school achievement of Amazonian Brazilian children. Moreover, these relationships will be examined based on both school achievement levels and the distance of the child's residence from the school to provide a nuanced understanding of this complex system.

## METHODS

### Population and context

This was a cross‐sectional study. The final sample comprised 106 elementary school students (2nd to 5th grade), aged 7 to 12 years, from a rural public school. The school, located in *Placas'* village (southeastern region of Pará, Brazilian Amazon), is the only one in the village to attend this age group.

This selection was intentional, aiming to investigate the unique and specific factors within this educational setting, a community whose unique structure is rooted in its historical origin as a resettlement area. This approach maximizes insights into the complex system operating in this unique environment.

The socioeconomic profile of the sample is predominantly low and medium, and the local economy relies on subsistence activities (agriculture, livestock and fishing). Although all students attend the same school, the sample includes children residing both in the village core and in distant rural properties distributed along the access roads to the village. This geographic stratification imposes significant logistical barriers: the distance from residence to school ranges from a few meters up to approximately 30 km (approximately 18.6 miles), resulting in commute times that can reach two hours in some cases, particularly during the rainy season due to unpaved roads. This unique combination of socioeconomic context and varied access conditions reinforces the importance of analysing the influence of environmental and geographical factors on school achievement outcomes.

### Data collection procedures

#### Sociodemographic information

The school provided student information on gender, date of birth and place of residence, following parental authorization. We organized residential data by locality name to categorize children based on their distance from the school into two groups: ‘village’ (≤1000 m) and ‘distant village’ (>1000 m) (approximately 0.62 miles).

This cutoff point was established based on a spatial visual analysis of the distribution map of the houses in the region. An observation of the housing clusters revealed a clear demarcation: Residences within the village core are concentrated within a 1000‐m radius surrounding the school, whereas the remaining residences, located along the access roads (distant village), are dispersed beyond this nucleus. Thus, this distance effectively categorizes the nucleated village versus the dispersed rural surroundings. For many in the ‘distant village’ group, the school is the educational pole for surrounding areas, with travel times reaching up to two hours. This demarcation, therefore, captures a fundamental difference in daily accessibility and interaction with the school environment.

#### Physical activity

The School Food Consumption and Physical Activity Questionnaire (CAAFE) was used to assess participants' physical activity (Costa et al., 2013). This web‐based interactive instrument was administered individually to all participants by a trained researcher who was present to clarify questions, minimize memory bias and ensure accurate recording of the data. The CAAFE collects information on activities performed on the preceding day, specifically segmenting the recall across the morning, afternoon and evening shifts. As demonstrated by previous research, the instrument possesses satisfactory validation and reproducibility, with results that resemble the precision of other comparable assessment methods (Jesus et al., 2016; Kupek & Liberali, 2024).

Based on these responses, metabolic equivalents (METs) were estimated using reference values from Butte et al. (2018). The total METs value for each child was then calculated as the sum of all estimated METs corresponding to the reported activities across the entire day.

#### Physical fitness

We assessed physical fitness following the guidelines *of Projeto Esporte Brasil* (PROESP‐BR) (Gaya et al., 2021). The evaluation included: (i) flexibility (sit‐and‐reach test); (ii) localized muscular endurance (1‐minute abdominal crunch test); (iii) lower limb power (horizontal jump test); (iv) upper limb power (2 kg medicine ball throw); and (v) agility (4 × 4‐m square agility test).

To create a comprehensive physical fitness score, adjusted for age and gender, we converted the raw test scores into z‐scores. Before calculating the global score, we multiplied the results of the agility test by (−1) to align their interpretation with the other test results.

#### Motor coordination

We evaluated motor coordination using the Motor Assessment Test for Children—KTK (*Körperkoordinationstest für Kinder*). The KTK comprises four tasks: (i) balance (balance beam); (ii) lower limb coordination (single‐leg jump); (iii) speed (lateral jumps); and (iv) spatial–temporal awareness and laterality (platform transfer) (Gorla et al., 2014; Kiphard & Schilling, 1974). After the tests, we aggregated the Motor Quotients (QMs) from each task to form a global motor coordination score.

#### Attention level

The Cancellation Attention Test was used to assess simple, sustained and alternating selective attention. Participants had to identify specific visual stimuli within a response matrix, with a one‐minute time limit for each task. We then computed the raw score for each task by subtracting the sum of errors and omissions from the number of correct responses. Subsequently, a global attention score was computed by aggregating the raw scores from all three tasks: simple selective, sustained and alternating attention (Montiel & Seabra, 2012).

#### School achievement

We employed the School Performance Test (TDE) to measure students' school achievement in three fundamental skills: writing, arithmetic and reading (Stein, 1994). Tests were administered in order of ascending difficulty, and the assessment for a student ended when they could no longer complete the presented task.

Subsequently, we summed the scores (writing + mathematics + reading = general school achievement) and categorized students into ‘good’ or ‘insufficient’ school achievement groups based on the median score.

### Statistical procedures

Data normality using the Kolmogorov–Smirnov test was assessed, along with skewness and kurtosis values. Data are presented as mean (standard deviation) and frequency (relative and absolute). We used independent samples *t*‐tests for comparative analyses based on school achievement and place of residence.

We then conducted a network analysis to explore the non‐linear relationships between variables, performing the analysis on four subgroups defined by school achievement (good and insufficient) and place of residence (village and distant village). Within these subgroups, we examined associations among age, attention levels, physical activity, motor coordination and physical fitness. For the residence subgroups, we also analysed variations in reading, writing and arithmetic abilities.

The networks were estimated using the GGM, which models the conditional partial correlations between the variables. Edge selection was performed using the graphical LASSO regularization method, which was optimized via the EBIC, with the regularization parameter set to 0.5, as recommended for obtaining stable models. This method ensures network parsimony by removing spurious connections.

We applied four centrality measures to understand each variable's role in the network, as described by Hevey (2018): betweenness, which highlights a node's importance in connecting other nodes; closeness, which reflects how quickly a node can impact or be impacted by others; strength, which indicates the intensity of a node's direct connections; and expected influence, which refers to a node's sensitivity to change and its function as a link between variable groups. A bootstrap with 1000 subsamples was simulated, consistent with previous research (Devecchi et al., 2022; Zarate et al., 2022).

We chose the partial correlation estimator for its ability to reveal connection strengths while controlling for the effects of other nodes in the model, which is similar to controlling for confounding effects in multiple regression (Hevey, 2018). The use of this estimator is supported by previous studies (Machado et al., 2021). All statistical procedures were performed using SPSS (version 25.0) and JASP (0.16.4), with a significance level of 95% (*p* ≤ .05).

### Ethical aspects

All participants received comprehensive information regarding the study. The research adhered to the Declaration of Helsinki and was approved by the Research Ethics Committee of the Federal University of Sergipe (5.155.350). Parents or legal guardians provided signed consent, and participating children provided signed assent, following the guidelines of the Brazilian Research Ethics Committee.

## RESULTS

Descriptive statistics and group comparisons are presented in Table 1. Children with better school achievement were significantly older (9.2 ± 1.0 vs. 8.0 ± 1.1; *p* < .001) and demonstrated higher levels of alternating selective attention (94.5 ± 22.7 vs. 79.8 ± 31.8; *p* = .01) than their peers with insufficient performance. This high‐achieving group also showed higher scores in balance (101.9 ± 7.8 vs. 97.4 ± 11.0; *p* = .05), lower limb coordination (81.2 ± 9.3 vs. 75.3 ± 11.4; *p* = .02) and global physical fitness (0.83 ± 4.1 vs. −1.33 ± 3.2; *p* = .01). No other significant differences were observed between achievement subgroups.

**TABLE 1:** Descriptive information of the variables considering school performance and place of residence.

Variables	School achievement				Place of residence			
	Good 45 (48.9%)	Insufficient 47 (48.9%)	*T*	*p*	Village 42 (39.6%)	Distant village 64 (60.4%)	*t*	*p*
Age (years)	9.2 ± 1.0	8.0 ± 1.1	−4.952	**<0.001**	8.0 ± 0.9	8.9 ± 1.1	−4.207	**<.001**
Physical activity	0.2 ± 0.9	−0.1 ± 1.0	−1.569	0.12	0.2 ± 1.0	−0.1 ± 0.9	1.579	.11
*Motor coordination*								
Balance	101.9 ± 7.8	97.4 ± 11.0	−1.882	**0.05**	98.7 ± 9.5	100.4 ± 10.0	−0.699	.48
Lower limb coordination	81.2 ± 9.3	75.3 ± 11.4	−2.378	**0.02**	77.7 ± 8.4	79.0 ± 11.5	−0.504	.61
Speed	86.7 ± 14.4	85.0 ± 14.4	−0.494	0.62	89.7 ± 11.5	83.2 ± 14.8	1.881	.07
Laterality	80.6 ± 10.7	79.7 ± 11.8	−0.241	0.62	82.7 ± 10.5	78.6 ± 11.8	1.423	.15
Global motor coordination	83.9 ± 8.6	79.7 ± 11.8	−1.716	0.91	83.8 ± 9.3	81.0 ± 11.0	1.086	.28
*Physical fitness*								
Flexibility	0.1 ± 1.1	−0.2 ± 0.9	−1.471	0.14	0.1 ± 1.0	−0.1 ± 1.0	0.033	.97
Localized muscle resistance	0.1 ± 1.2	−0.2 ± 0.7	−1.988	**0.05**	0.1 ± 1.0	−0.1 ± 0.9	0.588	.55
Lower limb power	0.1 ± 0.9	−0.1 ± 1.1	−1.123	0.26	0.1 ± 0.9	−0.1 ± 1.0	0.920	.36
Upper limb power	0.2 ± 1.0	−0.3 ± 0.9	−2.406	**0.01**	0.1 ± 0.9	−0.1 ± 1.0	1.126	.26
Agility	−0.1 ± 0.9	0.1 ± 1.0	0.612	0.53	−0.1 ± 0.9	0.1 ± 0.9	−0.902	.36
Global physical fitness	0.8 ± 4.1	−1.3 ± 3.2	−2.657	**0.01**	0.3 ± 3.6	−0.3 ± 3.7	0.791	.43
*Selective attention*								
Simple selective	106.6 ± 10.8	103.9 ± 20.8	−0.736	0.46	107.6 ± 11.5	104.9 ± 18.5	0.765	.44
Sustained selective	100.1 ± 33.0	91.0 ± 44.2	−1.070	0.28	92.0 ± 47.7	100.1 ± 35.8	−1.930	.06
Alternating selective	94.5 ± 22.7	79.8 ± 31.8	−2.457	**0.01**	87.2 ± 30.3	85.0 ± 29.2	0.214	.83
Global attention	104.0 ± 17.5	99.0 ± 19.7	−1.237	0.22	103.5 ± 16.5	100.6 ± 20.3	0.704	.45
*School achievement*								
Mathematics					5.7 ± 3.9	6.95 ± 3.4	−1.585	.11
Writing					4.5 ± 6.6	7.31 ± 7.9	−1.721	.08
Reading					14.9 ± 21.6	24.76 ± 24.6	−1.926	.**05**
Global school achievement					25.2 ± 30.9	38.45 ± 33.6	−1.882	.**05**

*Note*: Values presented as a mean standard deviation; Physical activity (age and sex‐adjusted *z*‐score); *t*, *t*‐test values for independent samples. Bolt values indicate significant differences (*p* ≤ .05).

Regarding the place of residence, children living closer to the school (‘village’ group) were significantly younger than those living farther away (8.0 ± 0.9 vs. 8.9 ± 1.1; *p* < .001). In terms of school achievement, students from the ‘distant village’ group had significantly higher global school performance scores than their peers in the ‘village’ group (38.45 ± 33.6 vs. 25.2 ± 30.9; *p* = .02).

Centrality measures for the school achievement and residence networks are shown in Tables 2 and 3, respectively. In the insufficient school achievement network, motor coordination was the central node, exhibiting the highest values for betweenness (2.13), closeness (1.85) and strength (1.49), while alternating selective attention had the highest expected influence (1.85). In the high school achievement network, age was the most central node across betweenness (1.33), closeness (1.41) and strength (1.20).

**TABLE 2:** Centrality measures according to school achievement.

Variables	School achievement							
	Betweenness		Closeness		Strength		Expected influence	
	Insuf.	Good	Insuf.	Good	Insuf.	Good	Insuf.	Good
Age (years)	−0.58	**1.33**	−0.94	**1.41**	−1.02	**1.20**	−0.80	−1.62
Simple attention	−0.13	−1.00	−0.04	−0.36	0.83	−0.48	−0.41	0.85
Sustained attention	−0.58	−1.00	−0.79	−0.24	−0.86	0.46	−0.56	−0.88
Alternating attention	−0.58	0.16	0.22	0.53	0.04	1.15	**1.85**	−0.35
Motor coordination	**2.13**	0.94	**1.85**	−1.80	**1.49**	−1.48	0.69	0.15
Physical activity	0.32	−1.00	0.54	0.53	0.52	−0.73	−0.96	0.79
Physical fitness	−0.58	0.55	−0.83	−0.06	**1.01**	−0.12	0.18	**1.05**

*Note*: Physical activity and physical fitness (*z*‐score adjusted for age and sex effect). Insuf. (Insufficient school achievement) and good (Good school achievement). Bolt values indicate prominent centrality measures in the network.

**TABLE 3:** Centrality measures according to place of residence.

Variables	Place of residence							
	Betweenness		Closeness		Strength		Expected influence	
	Village	Distant village	Village	Distant village	Village	Distant village	Village	Distant village
Age (years)	**1.94**	−0.53	**1.68**	−0.24	**1.70**	−1.03	−0.67	−0.87
Simple attention	−0.77	−0.91	−0.96	0.82	−0.77	0.52	−1.05	−0.81
Sustained attention	1.04	−0.15	0.35	0.41	0.37	−0.01	−0.26	−1.16
Alternating attention	−0.77	−0.15	0.42	0.39	0.33	0.80	−0.71	1.05
Motor coordination	−0.31	**1.36**	−1.07	−0.40	−1.53	−0.74	−0.60	0.92
Physical activity	−0.77	0.98	−0.93	**1.15**	−0.75	**1.16**	−0.80	−0.93
Physical fitness	−0.77	−0.91	−1.06	−1.96	−0.99	−1.71	0.63	−0.33
Writing	−0.77	**1.74**	−0.15	−0.30	0.02	0.49	0.30	**1.32**
Mathematics	**1.04**	−0.91	**1.22**	**1.18**	**0.94**	**1.19**	**2.00**	−0.37
Reading	0.13	−0.53	0.49	−1.05	0.67	−0.69	1.16	1.18

*Note*: Physical activity and physical fitness (*z*‐score adjusted for age and sex effect). Bolt values indicate prominent centrality measures in the network.

In the ‘village’ group network, age was again the most central node, while mathematics showed the highest expected influence (2.00). In the ‘distant village’ network, writing was the most influential node, with the highest betweenness (1.74) and expected influence (1.32). In this same network, mathematics and physical activity showed the highest closeness and strength values.

**FIGURE 1:**
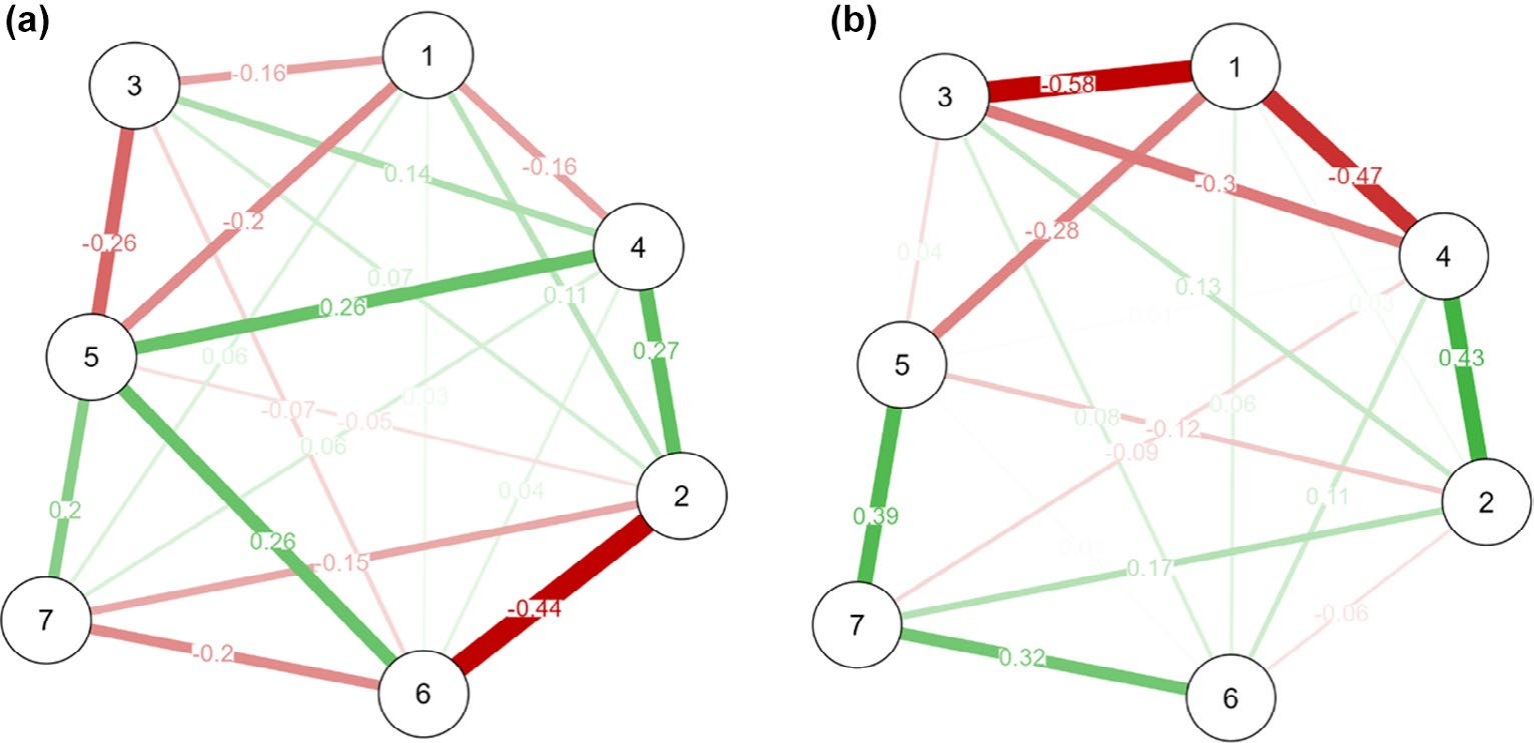
Weighting matrix network, according to school achievement. (a) insufficient achievement; (b) good achievement. (1) age; (2) selective attention; (3) sustained attention; (4) alternating attention; (5) motor coordination; (6) physical activity; (7) physical fitness. Green edges indicate positive partial correlations, while red edges indicate negative partial correlations. The thickness and saturation of the edges reflect the strength of the correlation coefficient; thinner/lighter lines represent weaker associations and thicker/darker lines represent stronger associations. Nodes (circles) represent the variables as defined in the legend.

**FIGURE 2:**
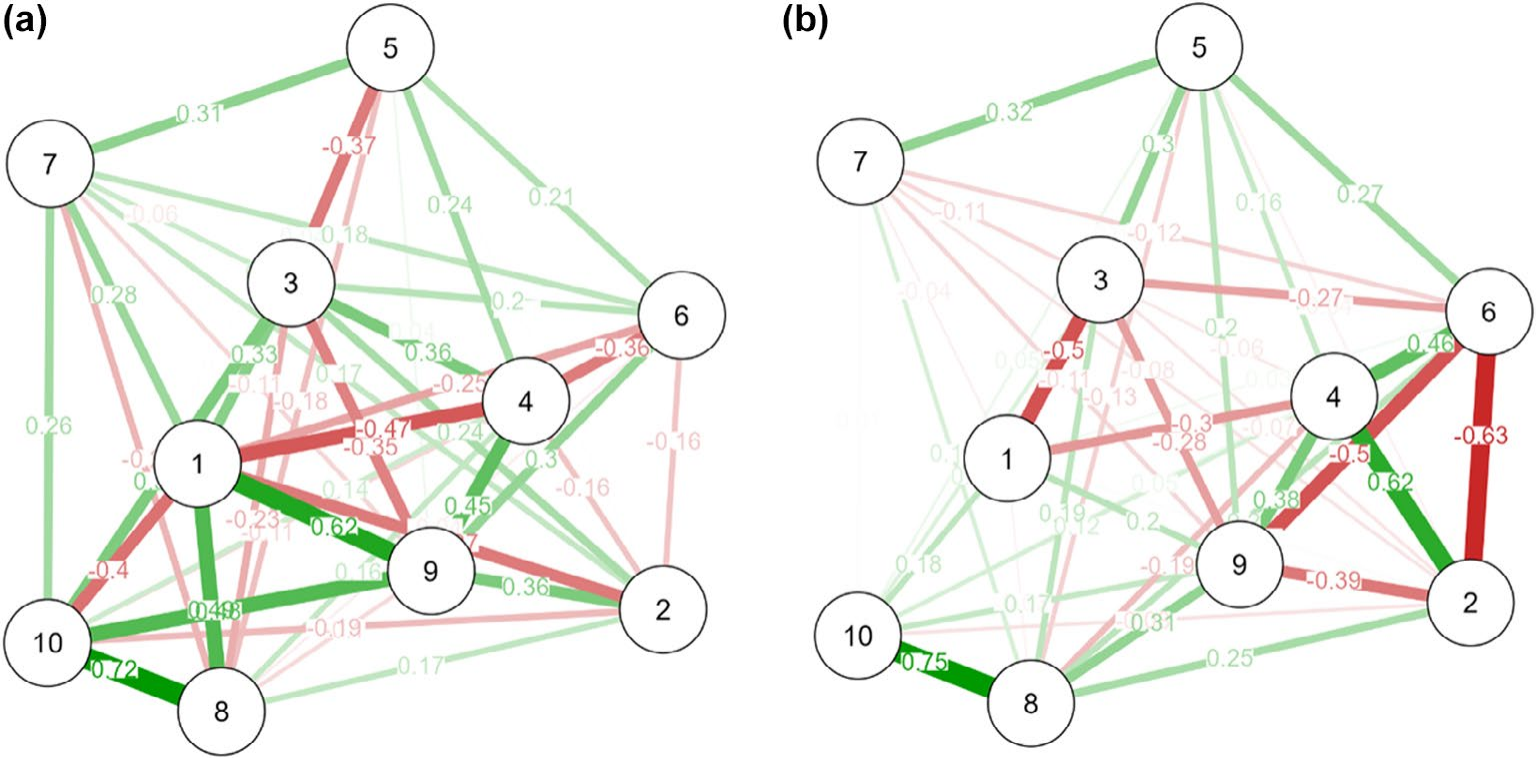
Weighting matrix network, according to place of residence. (a) village; (b) distant village. (1) age; (2) selective attention; (3) sustained attention; (4) alternating attention; (5) motor coordination; (6) physical activity; (7) physical fitness; (8) writing; (9) mathematics; (10) reading. Green edges indicate positive partial correlations, while red edges indicate negative partial correlations. The thickness and saturation of the edges reflect the strength of the correlation coefficient; thinner/lighter lines represent weaker associations and thicker/darker lines represent stronger associations. Nodes (circles) represent the variables as defined in the legend.

The network structures, illustrating the correlations (edges) between variables (nodes), are visualized in Figures 1 and 2. In the insufficient achievement group (Figure 1a), the strongest connection was a negative correlation between selective attention and physical activity (−0.44). In the high achievement group (Figure 1b), age was negatively correlated with sustained (−0.58) and alternating attention (−0.47), while physical fitness was positively correlated with motor coordination (0.39) and physical activity (0.32).

In the ‘village’ group network (Figure 2a), strong positive correlations were observed among the academic variables, particularly between reading and writing (0.72) and reading and mathematics (0.48). Age was also positively correlated with writing (0.49) and mathematics (0.62). In the ‘distant village’ network (Figure 2b), the strong positive association between reading and writing was maintained (0.62), along with a positive link between writing and mathematics (0.31). In summary, improvements in one of these variables had a positive impact on the other.

## DISCUSSION

This study employed a network analysis approach to investigate the complex interplay of individual and environmental factors on the school achievement of children in the Brazilian Amazon. Our primary findings revealed that: (i) students with better school achievement were older and scored higher in physical fitness, motor coordination and selective attention; (ii) contrary to general assumptions, students living farther from the school had significantly better global school achievement; and (iii) the network analysis identified distinct central variables for different subgroups, with age being central for high‐achievers, while motor coordination was central for those with insufficient achievement.

Consistent with a large body of literature, our results confirm a strong positive association between physical abilities and school achievement. We found that children with higher school achievement also presented better global physical fitness. This can be explained by the mediating role of physical activity. Enhanced physical fitness is often a direct result of regular engagement in physical activities, which are known to positively impact the development of executive brain functions (Fernandes et al., 2016). These functions, such as planning, working memory and cognitive flexibility, are crucial for both motor and cognitive tasks, including classroom activities (Lima et al., 2019).

Therefore, beyond its well‐established health benefits, regular physical activity is linked to improved cognitive functions that translate into better student behaviour and academic success, as supported by previous studies linking physical activity to performance in subjects like Portuguese and Maths (Bastos et al., 2015). The network analysis further supports this, showing a positive connection between physical fitness, motor coordination and physical activity in the high‐achieving group, suggesting a synergistic relationship where improvement in one area may bolster the others.

The analysis of age revealed a nuanced and complex role. As expected, age was a central node connecting other variables, particularly for children with good school achievement and those living closer to the school. This reflects a natural developmental cascade, where advancing age leads to maturation and improvement across various motor and cognitive skills (Gallahue et al., 2013). However, we also identified a counterintuitive negative association between age and attention in the high‐achieving group. This suggests that as children in this specific context grow older, they may experience greater fluctuations in attention or be more prone to mind‐wandering (Slattery et al., 2022). This unexpected finding warrants further investigation, as it may point to specific challenges faced by older students in this rural educational setting.

Perhaps the most striking and counterintuitive finding of this study was the negative relationship between proximity to school and academic performance. Our data clearly showed that children living farther from the school (‘distant village’ group) had significantly higher global school achievement scores. This finding directly challenges the common assumption that greater distance is a barrier to education. However, this result should be interpreted with caution, as this cross‐sectional study captures only an association, not a causal relationship, and it is potentially confounded by unmeasured distal variables.

While we did not collect data to fully explain this phenomenon, we can propose several hypotheses grounded in the unique context of this rural Amazonian setting. One possible explanation is an inverse ‘neighbourhood effect’. In urban settings, proximity to peers with poor academic histories can negatively impact a child's performance (Araujo & Silveira Neto, 2021). For the distant village group, the reduced residential proximity to peers may translate to fewer distractions and greater focus on homework and study time at home (Araujo & Silveira Neto, 2021). It is also possible that this relationship is confounded by unmeasured factors, such as specific parental educational involvement or family structures that provide unique academic support, which may be disproportionately present in the more remote vicinal areas, factors that were not fully captured by our socioeconomic status assessment (Antonelli‐Ponti et al., 2021; Hossain et al., 2021).

In this sparsely populated rural context, living farther away may translate to fewer social distractions and more structured time at home, creating a more conducive environment for homework and study. Furthermore, unmeasured variables, such as the socioeconomic profile of families living on more distant properties or a higher degree of parental involvement necessitated by the logistics of rural life, could also be contributing factors. This finding underscores the critical importance of understanding local context, as factors that act as barriers in one environment may not be in another.

The present findings, interpreted through the lens of network centrality, provide valuable insights for identifying potential priority areas for developing school‐based programs. Given the study's cross‐sectional design, these are suggested focal points based on association and network structure, not direct causal recommendations, but they may guide strategies aimed at improving student achievement. Considering the identified associations, some actions may be considered for the development of each factor.

Considering that motor coordination ranked as the node with the highest overall centrality, strongly connecting with other variables for the insufficient achievement group, physical education classes could offer means for children to enrich their motor repertoire, enhancing it further. This suggests that motor skill development may represent a potential leverage point within the complex system influencing school achievement in this vulnerable subgroup (Vilella‐Cortez et al., 2019).

Better development of physical fitness skills reduces the likelihood of motor problems in children (Chaves et al., 2016), and promoting proper motor coordination may not only improve physical health but also positively impact attention and engagement in school activities (Souza Neto et al., 2021; Zanella et al., 2016), thereby preventing further academic difficulties. By contrast, for children in the ‘distant village’ group, who performed better academically, writing ability was the most central node. This highlights the foundational role of literacy skills, which act as a gateway to success across all academic domains, as these skills are deeply interconnected with reading and mathematics (Assari et al., 2021; Athayde et al., 2019).

This study has limitations that must be acknowledged. Its cross‐sectional design prevents us from making causal inferences. Additionally, specific limitations apply to the network analysis performed on segregated subgroups. Given the resultant small sample size of the subgroups, the centrality indices presented should be interpreted cautiously as measures of relative importance and structure specificity within that particular group, rather than definitive, absolute rankings of centrality. Although the reliability of the centrality estimates was empirically confirmed through the bootstrap procedure, the findings are structural and not intended for broad generalization outside the defined model. Moreover, the use of METs values to estimate physical activity is a limitation.

Despite these limitations, this research makes a significant methodological contribution by being one of the few studies to analyse school achievement in a rural Amazonian context using a holistic approach that integrates individual, environmental, cognitive and motor factors. A pivotal strength of this investigation resides in its unique sample selection. Our research includes children from a rural context in the Brazilian Amazon, deliberately deviating from the pervasive sampling bias found in developmental science literature. This methodological decision enhances the global relevance of our findings, as it allows the mapping of developmental and academic mechanisms within an environment characterized by specific structural and logistical constraints. The results offer context‐specific insights that are vital for formulating globally sensitive interventions, thereby contributing significantly to better representing the diversity of human development across varied environmental settings.

## CONCLUSION

Children with better school achievement in this rural Amazonian context were typically older and displayed superior concentration, enhanced motor coordination skills and higher overall physical fitness. On the opposite to general assumptions, students living farther from school demonstrated better school achievement, a finding that highlights the unique influence of the local environment. The interconnectedness of fundamental academic skills, such as reading, writing and math, was also confirmed.

The analysis identified stimulating motor coordination as a valuable strategy for improving school achievement, especially among students with insufficient performance. Overall, these findings underscore that academic success is a complex interplay of individual and environmental factors, suggesting that effective interventions must look beyond cognitive aspects to also include motor and behavioural development.

## AUTHOR CONTRIBUTIONS

**Douglas Vieira:** Conceptualization; investigation; writing – original draft; methodology; validation; writing – review and editing; formal analysis; project administration; supervision. **Cinthia Rodrigues Barros:** Writing – review and editing. **Mabliny Thuany:** Writing – original draft; writing – review and editing; formal analysis; supervision. **Thayse Natacha Gomes:** Conceptualization; writing – original draft; writing – review and editing; supervision; formal analysis; project administration; methodology; validation; investigation.

## FUNDING INFORMATION

This work was supported by the Brazilian Federal Agency for Support and Evaluation of Graduate Education.

## CONFLICT OF INTEREST STATEMENT

The authors declare that there is no conflict of interest.

## ETHICS STATEMENT

The Research Ethics Committee of the Federal University of Sergipe approved this study (reference number 5.155.350).

## Data Availability

The data that support the findings of this study are available from the corresponding author upon reasonable request.
